# Music and Dyslexia: A New Musical Training Method to Improve Reading and Related Disorders

**DOI:** 10.3389/fpsyg.2016.00026

**Published:** 2016-01-22

**Authors:** Michel Habib, Chloé Lardy, Tristan Desiles, Céline Commeiras, Julie Chobert, Mireille Besson

**Affiliations:** ^1^Résodys et Service d'Éducation Spéciale et de Soins à Domicile Résodys, Agence Régionale de la SantéMarseille, France; ^2^Laboratoire de Neurosciences Cognitives, Centre National de la Recherche Scientifique and Aix-Marseille UniversitéMarseille, France

**Keywords:** dyslexia, music therapy, phonology, reading, attention, learning disorders

## Abstract

Numerous arguments in the recent neuroscientific literature support the use of musical training as a therapeutic tool among the arsenal already available to therapists and educators for treating children with dyslexia. In the present study, we tested the efficacy of a specially-designed Cognitivo-Musical Training (CMT) method based upon three principles: (1) music-language analogies: training dyslexics with music could contribute to improve brain circuits which are common to music and language processes; (2) the temporal and rhythmic features of music, which could exert a positive effect on the multiple dimensions of the “temporal deficit” characteristic of some types of dyslexia; and (3) cross-modal integration, based on converging evidence of impaired connectivity between brain regions in dyslexia and related disorders. Accordingly, we developed a series of musical exercises involving jointly and simultaneously sensory (visual, auditory, somatosensory) and motor systems, with special emphasis on rhythmic perception and production in addition to intensive training of various features of the musical auditory signal. Two separate studies were carried out, one in which dyslexic children received intensive musical exercises concentrated over 18 h during 3 consecutive days, and the other in which the 18 h of musical training were spread over 6 weeks. Both studies showed significant improvements in some untrained, linguistic and non-linguistic variables. The first one yielded significant improvement in categorical perception and auditory perception of temporal components of speech. The second study revealed additional improvements in auditory attention, phonological awareness (syllable fusion), reading abilities, and repetition of pseudo-words. Importantly, most improvements persisted after an untrained period of 6 weeks. These results provide new additional arguments for using music as part of systematic therapeutic and instructional practice for dyslexic children.

## Introduction

There is worldwide agreement for estimating between 5 and 15% the school-age population that fails to get into initial learning that is, to acquire reading, writing, and/or calculation correctly, despite normal intelligence and in the absence of gross psycho-affective or socioeducative deficiency. This deficit corresponds to the “specific learning disorder” section of the latest international classification (DSM-5, 2013). Among these disorders, dyslexia has been the subject of numerous studies in recent years with results clearly demonstrating functional and structural brain abnormalities from both genetic and cultural origins. In short, experiments using brain imaging have shown abnormal activation in several cortical and subcortical brain regions and cerebellum (Démonet et al., 2004) as well as a lack of connectivity between these different areas in children or adults with dyslexia (Finn et al., 2014). This last group of results opens promising new perspectives for understanding the mechanisms underlying dyslexia and related disorders, as well as to guide remediation (van der Mark et al., 2011; Vandermosten et al., 2012). For instance, results of a recent study combining multiple imaging methods (functional magnetic resonance imaging—fMRI—, functional and structural connectivity) revealed that phoneme discrimination impairments, one of the halmarks of cerebral dysfunction in dyslexia, reflect a failure to access otherwise intact phonemic representations via the subcortical white matter bundles (including the so-called “arcuate fasciculus,” which links Broca's area to the temporo-parietal regions; Boets et al., 2013). The direct implication of this finding is that rehabilitation methods of dyslexia should not only focus on restoring phonological representations, as is the case of most remediations currently used with these children, but also on restoring functional connections between frontal and temporal language areas. More generally, rehabilitation should aim at increasing the integration of information typically processed by different brain areas. As we will argue below, one way to reach this aim is through music training.

Active research in the domain of the neuroscience of music has demonstrated that the brain of professional musicians is an excellent model of brain plasticity (e.g., Münte et al., 2002) both at the subcortical and cortical levels (e.g., Kraus and Chandrasekaran, 2010; Besson et al., 2011). The musician's brain is ideally suited to study brain changes induced by intensive training and the effects of a targeted and repeated cognitive activity on brain morphology, as suggested for the rehabilitation of dyslexia (Keller and Just, 2009). Interestingly, some white matter subcortical tracts, including the arcuate fasciculus mentioned above and long-known as a crucial element within the left hemisphere language network, are particularly sensitive to learning to play a musical instrument or singing (Halwani et al., 2011), both skills requiring intense and fine coordination between sensory (visual, auditory, and somatosensory) and motor processes. Previous results also demonstrated structural differences in interhemispheric fibers of the anterior region of the corpus callosum connecting motor cortical regions of the right and left hands (e.g., Schlaug et al., 1995). This finding may be related to structural abormalities of such interhemispheric fibers in children and adults with dyslexia, suggesting that inter-hemispheric communication failure may be one of the possible mechanisms underlying this disorder (Welcome and Joanisse, 2014).

Using musical training for the remediation of dyslexia and language disorders is based on both theoretical considerations and experimental results. If there are common underlying processes between music and language, especially between music perception and speech perception, one might assume that improving some of the processes involved in the perception of music can also improve speech perception and reading skills (e.g., Goswami et al., 2002; Patel, 2003, 2012; Kraus and Chandrasekaran, 2010; Besson et al., 2011; Corrigall and Trainor, 2011). In one of the first studies aimed at testing this hypothesis, Overy (2000, 2003) proposed a series of music games gradually increasing in difficulty and focusing on pace and “timing” skills to dyslexic children over a period of 15 weeks. Results showed significant improvements, not in reading skills, but in two related areas: phonological processing and spelling. More recently, Cogo-Moreira et al. (2012, 2013) reported that musical training had positive effects on reading skills and educational achievement in children and adolescents with dyslexia and Weiss et al. (2014) showed that adult musician dyslexics performed better than non-musician normal readers on various pitch interval discrimination tasks, finger rhythmic tapping, and speech in noise perception tasks.

The importance of word metric structure and, specificially, rise-time perception for speech processing has been stressed by Goswami et al. (2002). They proposed that misalignments between neuronal excitability fluctuations in the auditory regions and maximum amplitudes in the speech signal may be related to phonological disabilities in children with dyslexia (Power et al., 2013). In line with this view, Bishop-Liebler et al. (2014) recently reported that adult musician dyslexics were better than non-musician dyslexics on various tests of temporal auditory processing and specifically for processing temporal envelope and “rise time.” In addition, musician dyslexics outperformed their non-musician peers on reading scores and also, to a lesser extent, on phonological awareness. Similarly, Flaugnacco et al. (2014) showed that, among other rhythm production and perception tasks, the level of performance on a metric perception task (i.e., perceiving changes in note duration within recurrent series) specifically predicted both reading speed and accuracy as well as phonological processing in Italian dyslexics. The authors concluded that their results strongly encourage the use of music training in dyslexia rehabilitation, and specifically recommended to “focus on rhythm rather than on pitch accuracy as is often the case in classical music pedagogy.” This recommendation is in line with recent work from the Kraus group (Slater et al., 2013), examining the effect of 1 year musical training based on the perception of pitch, rhythm (tapping in synchrony with a given tempo) and improvisation. The level of performance of 8 year-old children considered “at risk” for learning disability and who received this musical training was significantly higher than matched controls in the synchrony tapping task. Going one step further, Przybylski et al. (2013) examined the influence of rhym perception on syntactic processing. They presented to language and reading impaired chidren a rhythmic prime (a succesion of notes played either regularly or irregularly), immediately followed by a spoken sentence that was syntactically correct or incorrect (e.g., “Laura *has/have* forgotten her violin”). Results showed a clear superiority for regular over irregular rhythmic primes on the chidren's performance in the syntactic task. Based on these results, the authors proposed to use rhythmic stimulation in remediation protocols designed for chidren with oral and written language developmental disorders (see also Cason and Schön, 2012; Cason et al., 2015, for similar results with prelingually deaf children).

In a previous work from our group (Chobert et al., 2012), we recorded the Event-related brain Potentials in an original mismatch negativity (MMN) protocol to test for the pre-attentive perception of syllables varying in pitch, duration or voice-onset time. We found that dyslexic children differed from matched controls in the MMN to voice-onset time and to duration but not to pitch variations; that is, to the two time-related among the three variables examined. Directly related to this issue, Bidelman et al. (2014) conducted a series of experiments examining categorical perception, the cornerstone of speech perception, in younger and older adult musicians and non-musicians. Results consistently showed improved categorical perception in musicians than in non-musicians in both younger and older adults. Based on these results and others showing that categorical perception is often impaired in children with dyslexia (Serniclaes et al., 2004), the first aim of the experiments reported here was to further test for categorical perception of voice-onset time (identification and discrimination) in children with dyslexia compared to control normal-readers.

The second aim was to examine perception of word metric structures in children with dyslexia and in normal-readers. We used the materials built by Magne et al. (2007) and comprising trisyllabic words spoken at a normal speech rate or with an unusual lengthening of the penultimate syllable. We hypothesized that children with dyslexia would perform lower than normal readers in the detection of unusual syllabic lengthening.

The third aim was to further test pitch discrimination in dyslexic children since results reported in the literature are quite variable showing both normal or abormal pitch processing in different studies (e.g., Baldeweg et al., 1999; Santos et al., 2007; Chobert et al., 2012). We included a pitch discrimination task with changes in melodic contour, harmony or both contour and harmony in simple nursery rhymes.

The last and most important aim was to determine whether a specifically-designed Cognitive-Musical Training (CMT) method can improve categorical perception as well as perception of the metric structure of words and possibly pitch discrimination in children with dyslexia. Based on the literature review above, we reasoned that the CMT method should include at least three components: (1) an auditory component targetting the language-music similarity in auditory perception, (2) a motor component, mainly focusing on rhythm production and imitation, and (3) a cross-modal component, making special demands on simultaneous processing of information from different modalities including auditory, visual, sensory, and motor modalities as well as their combinations. To this end and in order to stimulate auditory attention and working memory abilities, the CMT program comprises a battery of musical tasks and exercises based on these components and on the active listening to various sorts of musical stimuli. Multimodal training combining different modalities, as is typically the case in real life, was achieved through tasks that simultaneously involve visual, auditory, and sensory-motor processing. Simplified visual and gestural supports were provided that were adapted to each child's level of performance. For instance, a very simple musical notation system was purposefully devised, made of only 3 or 5 strokes to represent pitch and duration of sounds. The rhythmic aspect of each task was emphazised (for details regarding the content of exercises and tasks, see the Methods Section below and Habib and Commeiras, 2014).

Two experiments were conducted with two different populations of dyslexic children, using similar tasks and materials and only differing in the duration of the musical training period. Thus, we compared the effects of the CMT program when training sessions were clustered on 3 consecutive days and when they were distributed over a period of 6 weeks.

## Study 1: Intensive cognitive-musical training (CMT) clustered on 3 consecutive days

### Methods

#### Participants

A group of 12 children from 8.2 to 11.7 years (mean 10 years 7 months, s.d. = 17 months) participated in the study. They all received a common diagnosis of severe dyslexia leading to their admission in specialized classes with multi-disciplinary support for dyslexic children. Thus, all children were already involved in intensive conventional rehabilitation methods, but during this 3-day CMT period, they did not receive speech therapy. The clinical characteristics of the children are reported in Tables 1, 2. A reading-age matched normal-reading group of 22 children (30 months younger on average), served as a control population for normative data. As seen from Tables 1, 2, the diagnosis of severe dyslexia rests on the presence of significant delay in terms of reading age as well as reading isolated words. General intelligence was largely preserved, as shown by scores on the similarities and matrices substests of the WISC-IV scale. Spelling and auditory-verbal short-term and working memory were more variably altered.

**Table 1:** **Level of performance of dyslexics and control normal-readers (with indication of two standard deviations below norm) in several standard psychometric tests**.

		**Dyslexics**		**Controls**	
		**Mean**	**±SD**	**Mean**	**−2 SD**
Reading age		90.0	±19.0	128.5	93.9
Oral and written language battery	Phonetic fluency	15.0	±2.8	15.0	8.0
	Semantic fluency	27.3	±9.0	23.5	16.0
	Hard words repetition	23.2^*^	±5.0	29.5^*^	26.0
	Reading strategy				
	Pseudowords	15.3^*^	±2.4	20.0^*^	16.0
	Regular	9.1	±1.2	10.0	9.4
	Irregular	6.8^*^	±1.9	10.0^*^	8.0
	Spelling				
	Phonological errors	11.3^*^	±3.0	15.0^*^	12.0
	Grammatical errors	10.0	±7.0	9.5	4.5
	Use rules errors	14.0	±5.9	18.5	9.5
WISC-IV	Similarities	10.4	±2.4	9.7	4.9
	Matrices	10.1	±3.0	10.3	4.3
	Digit span				
	Direct	7.8	±2.1	9.9	3.5
	Reverse	7.9	±3.4	9.9	4.7
	TOTAL	7.9	±2.6	9.9	4.5

*Significant control/patient differences are in bold (T-test*,

* *p < 0.05). Reading ages are in months*.

**Table 2:** **Dyslexia severity (age in month)**.

**Dyslexics**	**Chronological age**	**Reading age**	**Difference**
1	135	111	−24
2	133	90	−43
3	146	111	−35
4	146	88	−58
5	99	55	−44
6	97	77	−20
7	125	84	−41
8	112	58	−54
9	141	101	−40
10	124	106	−18
11	123	96	−27
12	145	114	−31
Mean	127	90	−36
*s.d.*	17	19	12

### Training procedure

#### Description of the CMT method

The CMT method was designed by speech therapists based on widely recognized principles of effective intervention (i.e., goal-directed, systematic, and coherent progression along a hierarchical structure; Shaywitz, 2005). Several exercises were built that covered various dimensions and components of music: pitch, duration, tempo, pulsation, and rhythm and that aimed at developing both the perception and the production sides. Exercises also comprised both sensory (visual and auditory) and motor components, engaging the child into transcoding processes from one modality to another (e.g., tapping in synchrony with a heard sequence, tapping the written notation of a rhythm; learning to play a small melody and to correct errors in other children's performance; etc… ). The use of a piano keyboard was systematically added to provide children with the visuo-spatial organization of the white and black keys, as a reinforcement for the sequential nature of the musical scale. Also and as often as possible, exercises required body movements to be performed in line with the musical excerpts. Finally, the connection between music and language was used through exercises implicating both speech and music (e.g., nursery rhymes, tracing the prosody of a sentence on a sheet of paper…). During the CMT sessions, no speech therapy was used and no conventional exercises (e.g., related to phonology reading or writing) were performed. However, most children were also involved in their usual weekly treatment, more or less half an hour, one or two times per week with a speech therapist.

The CMT program started on the first day of winter vacation in an outbuilding of the University Hospital, after the children and their legal representatives had agreed to participate in this experiment that was approved by the *ad-hoc* local ethics committee. They were fully informed of the aim and content of the research program. The training workshop lasted for 3 whole days, 6 h per day, for a total of 18 h. Children were divided into three groups of four and they all participated in three training sessions including (1) specific musical exercises given by a speech therapist, (2) music education with piano instruction with a piano professor, and (3) percussion and rhythmic bodily exercises with a psychomotor therapist. Each session lasted 45 min, with a 15 min break before moving onto the next training session. Each of the 3 days included the same sequence of sessions, only varying in level of difficulty. At the end of each day, all children met in a dance hall, where they practiced folk dancing with a specialized teacher. Children were informed that they will perform in front of their parents and teachers at the end of the third and final day to give a more recreational and challenging aspect to the whole training and to increase their motivation.

### Assessment tests

As described below, three tasks tapping into different aspects of auditory and speech perception, were used: categorical perception (identification and discrimination tests), syllabic duration and pitch variations. The level of performance in each task was measured in children with dyslexia both before and after training. For comparison purposes, the level of performance in each task was also measured in control normal-readers who did not follow the CMT program as they were not impaired in the perceptual, cognitive, and motor abilities that the CMT aimed at improving.

### Categorical perception of the phoneme “b” in the syllable [Ba]

#### Identification task

A 9-step continuum between two phonemes (i.e., “b” in syllable “Ba” from B1 to B4 and “p” as in syllable “Pa” from B5 to B9) was used with voicing delays varying between −52 and +20 ms. The identification rate of Ba (that of Pa being represented by the reverse curve) was computed.

#### Discrimination task

Eight pairs were formed (from Ba1–Ba2 to Pa8–Pa9) using the nine syllables of the continuum. The rate of correct discrimination (same-different) was computed.

#### Syllabic duration task

Children listened to 42 tri-syllabic words (e.g., “canapé”) and they had to decide whether the word was spoken normally or with an incongruous lenghtening of the penultimate syllable (e.g., “ca**naa**pé”). The percentage of correct responses was measured. **Pitch discrimination task**: children listened to five nursery rhymes played on the piano (e.g., “Sur le pont d'Avignon”; each around 20 s duration) that were recorded in four different versions: exact version, pitch change within the melodic contour, pitch change out of melodic contour, pitch change out of melodic contour and out of harmony, for a total of 20 trials. Children were asked to decide whether each fragment was the normal version or not.

### Data analysis

First, we compared dyslexics and normal-readers in the different tasks (identification and discrimination tasks for categorical perception, metric, and pitch discrimination tasks) using Analyses of Variance (ANOVAs) including Group (Dyslexics vs. Controls) as a between-subject factor and Position on the continuum (nine Positions; identification task), Pairs (eight different pairs; discrimination task), Syllabic duration (normal vs. lengthened; metric task), or Pitch (normal pitch, pitch change preserving melodic contour, pitch change out of melodic contour, and out of harmony) as within-subject factors in separate ANOVAs. To test for the effects of the CMT program in dyslexic children, we also computed ANOVAs including Session (before vs. after CMT) as well as the within-subjects factors described above for each task. Simple effects were analyzed using *post-hoc* Fischer's PLSD tests. All analyses were computed using the *Statistica* program.

### Results

#### Categorical perception

**Figure 1:**
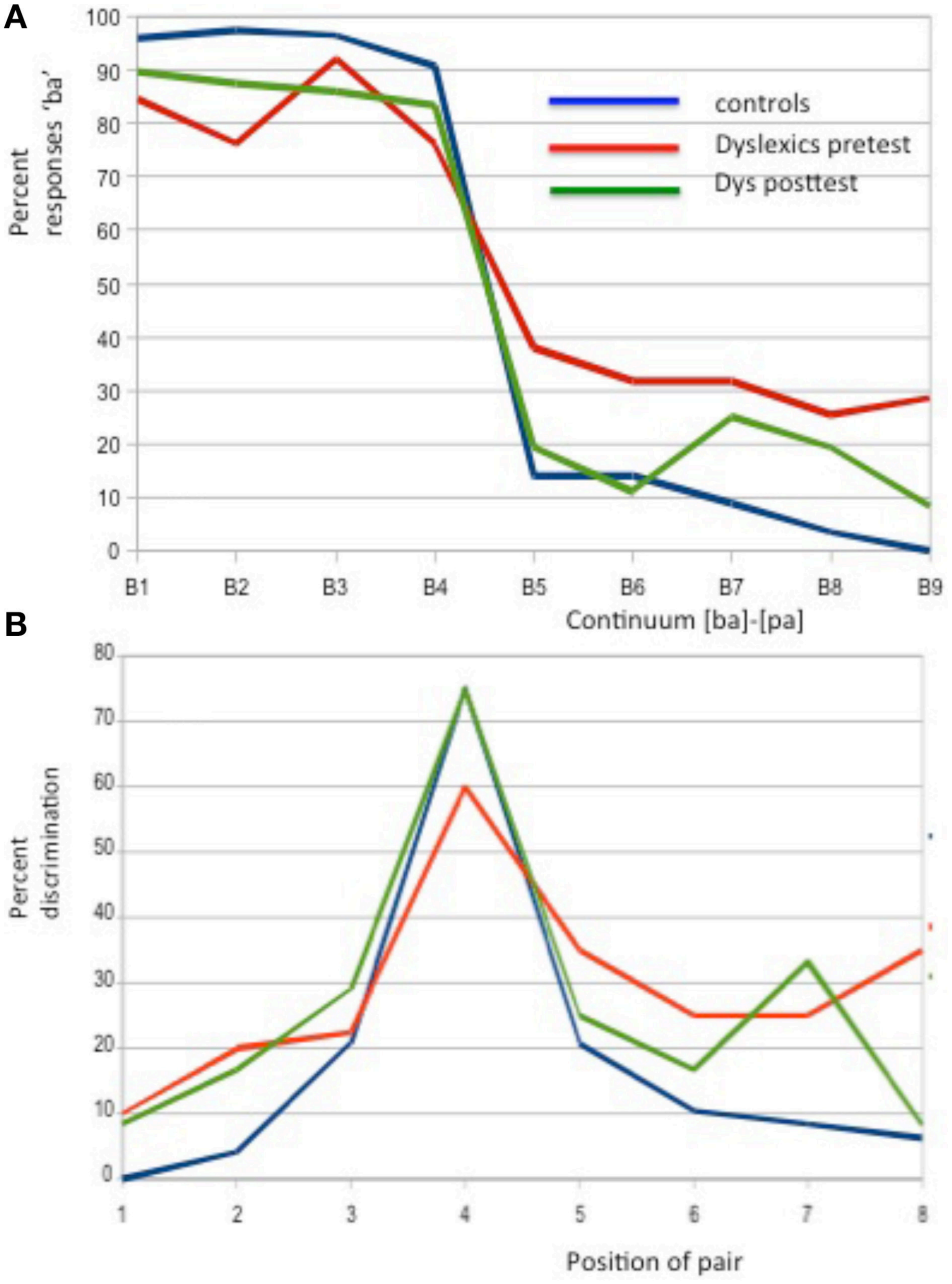
**Categorical perception using a nine steps continuum between the syllables [ba] and [pa]**. In the identification test **(A)** dyslexic children before CMT (red) showed less steep intercategorical boundary than normal readers (blue) but a “normalization,” specifically for B5 and B6 after CMT (green). In the discrimination task **(B)**, dyslexics before CMT (red) seemed to differ from normal readers (blue) for items at or close to the inter-categorical border (median peak in the figure) but these differences vanished after CMT (green).

As shown in Figure 1A, the percentage of syllables identified as “Ba” differed between children with dyslexia before CMT and normal-readers. However, while, the main effect of Group was not significant [*F*_(1, 29)_ = 2.16, *p* = 0.15], the main effect of Position [*F*_(8, 29)_ = 193.61, *p* < 0.001] and the Group × Position interaction [*F*_(8, 232)_ = 6.97, *p* < 0.001] were significant. *Post-hoc* analyses showed that the between-group differences were significant for position B2 (*p* < 0.007), with a higher percentage of “Ba” identification for dyslexics than controls as well as for B5 (*p* < 0.03), B8 (*p* < 0.02), and B9 (*p* < 0.002), with a lower percentage of “Ba” identification for dyslexics than for controls.

For the discrimination task (see Figure 1B), both inter- and intra-categorical pairs were presented (the latter being harder to discriminate). Neither the between-group difference (*F* < 1) nor the Group × Pairs interaction (*F* < 1) were significant. Only the main effect of Pairs was significant [*F*_(7, 33)_ = 5.56, *p* < 0.001] with the highest percentage of correct discrimination for the B4–B5 pair (Pair 4 on Figure 1B) for all children.

Testing for the effect of the CMT program, results of separate ANOVAs for dyslexics in the identification task showed significant improvements after 3 days of CMT with no main effect of Session (*F* < 1) but a significant main effect of Position [*F*_(1, 11)_ = 68.94, *p* < 0.001] and a significant Session × Condition interaction [*F*_(8, 88)_ = 2.41, *p* < 0.021]. *Post-hoc* analyses showed that both within-category and inter-category perceptions were modified after training (B2: *p* < 0.03; B6: *p* < 0.03; and B9: *p* < 0.04).

Results for dyslexics in the discrimination task revealed that the main effect of Session was marginally significant [*F*_(1, 11)_ = 3.61, *p* < 0.08] but the main effect of Pairs as well as the Session by Pairs interaction were significant [*F*_(1, 11)_ = 16.32, *p* < 0.001 and *F*_(7, 77)_ = 3.28, *p* < 0.004]. The improvement in phonetic discrimination after CMT was largest for the pairs 4 and 5. *Post-hoc* analyses confirmed both intra (pairs B1-B2; *p* < 0.02) and inter (B5-B6, *p* < 0.007) category perception improvement.

#### For the syllabic duration task (Figure 2)

The level of performance of dyslexics before training was significantly lower than for normal readers [main effect of Group: *F*_(1, 33)_ = 4.97, *p* < 0.03]. Moreover, all children performed lower for words with lengthening of the penultimate syllables than for normally spoken words [main effect of Condition: *F*_(1, 33)_ = 27.21, *p* < 0.001] and the Group × Condition interaction was also significant [*F*_(1, 33)_ = 4.2, *p* < 0.05]. *Post-hoc* comparisons showed that dyslexics (52%) performed lower than controls (64%) for words with lengthening of the penultimate syllables with no differences for normally spoken words (dyslexics: 73% and controls: 78%).

**Figure 2:**
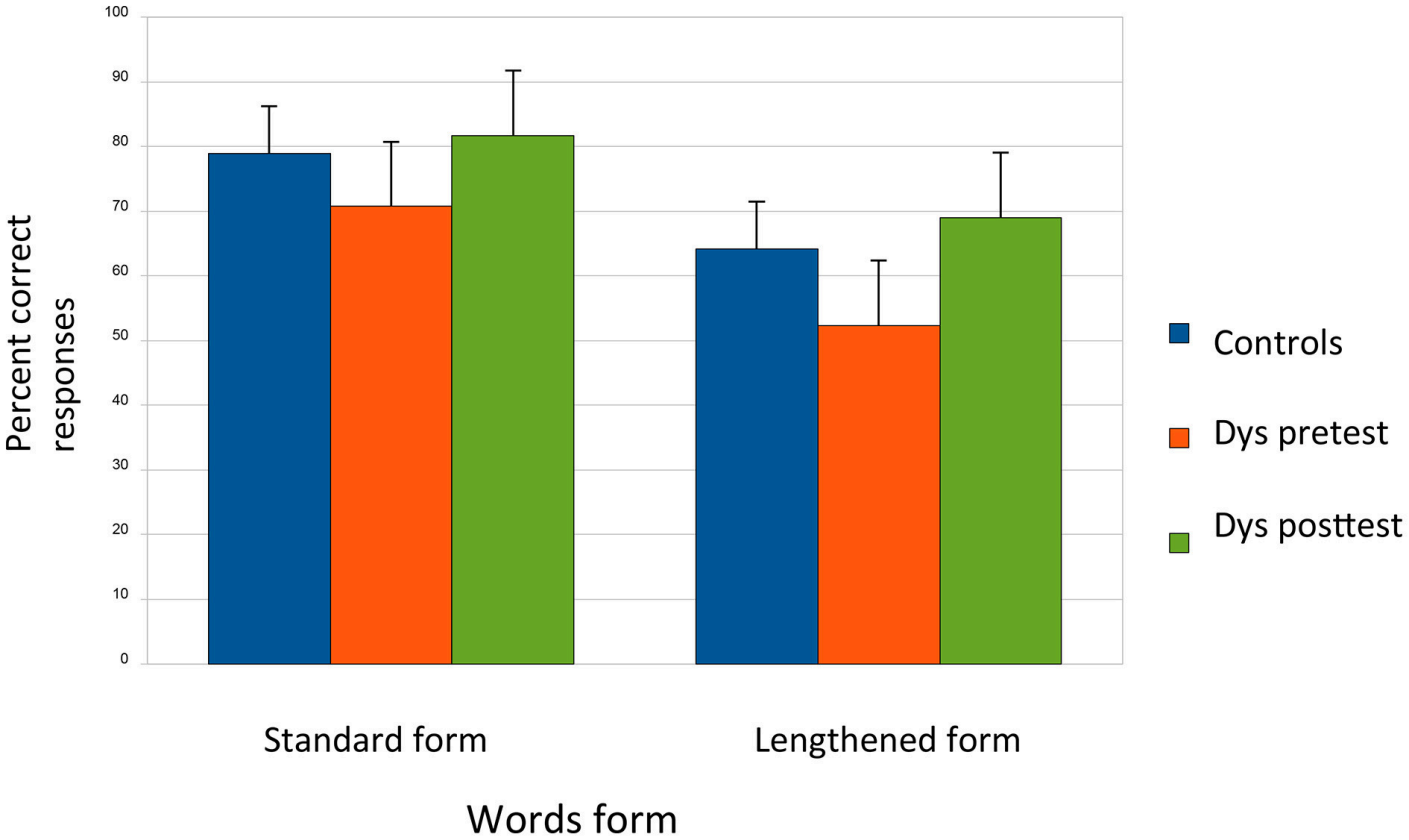
**Syllabic duration task: before CMT, dyslexics (red) performed lower than controls (blue) for words with an unusual lengthening of the penultimate syllables**. After the CMT program (green), the dyslexic's level of performance was higher for both type of words with stronger improvements for lengthened words.

Importantly, results of separate ANOVA for dyslexics, showed that the main effects of Session and Syllabic duration were significant [*F*_(1, 11)_ = 16.62, *p* < 0.001 and *F*_(1, 11)_ = 8.96, *p* < 0.01 respectively]. The Condition × Session interaction was only marginally significant [*F*_(1, 11)_ = 3.15, *p* = 0.10]: the level of performance of dyslexics was higher after CMT than before for both normally spoken words (after: 81% and before: 73%) and for lengthened words (after: 69% and before: 52%). Nevertheless, results of *post-hoc* tests showed that the improvement was larger for lengthened words (*p* < 0.002) than for normally spoken words (*p* < 0.02).

#### For the pitch discrimination task (nursery rhymes)

Results revealed that dyslexics did not differ from controls before CMT [main effect of Group: *F*_(1, 32)_ = 1.36, *p* > 0.25]. The main effect of Condition was significant [*F*_(3, 96)_ = 89.35, *p* < 0.001] but the Group × Condition interaction was only marginally significant [*F*_(3, 96)_ = 2.34, *p* = 0.07]. The differences between dyslexics and controls were larger for the exact version condition (*p* < 0.05) than for the other three conditions (all *p* > 0.50).

Results of separate ANOVA comparing dyslexic children before and after the CMT program showed no main effect of Session (*F* < 1) and no Session × Condition interaction (*F* < 1). The overall percentage of correct responses of dyslexic children was not higher after (56, 30, 26, 30%) than before CMT (54, 30, 30, 29%). By contrast, the main effect of Condition was significant [*F*_(3, 33)_ = 18.06, *p* < 0.001]. Results of *post-hoc* tests showed that the level of performance was highest for the “normal” condition (*p* < 0.002).

### Discussion

Results of this first experiment that aimed at testing the effects of an intensive use of the CMT method over 3 consecutive days in children with dyslexia, revealed two findings of main interest. First, compared to normal-readers, dyslexics were impaired in the identification test of categorical perception but their level of performance reached the level of control children after 3 days (18 h) of the CMT program. That dyslexics and controls were significantly different in the identification task before training suggests an excessive intra-categorical and less clear inter-categorical perception. Importantly, intensive music training positive influenced categorical perception by facilitating syllabic identification based on differences in VOT between the “b” and “p” phonemes. Likewise, there was a significant improvement in the discrimination test of categorical perception wherein intra-categorical pairs were more often perceived as different by dyslexics than by normal-readers, possibly reflecting some type of allophonic perception (Serniclaes et al., 2004). Overall, these results are in line with improved perception of VOT with music training in normal readers (e.g., Chobert et al., 2014) and with vowel identification in young and older musician compared to non-musician adults (Bidelman et al., 2014).

The present results also showed that while the level of performance of children with dyslexia before CMT was lower than normal readers in the syllabic lengthening task, it was significantly improved after 3 days of CMT. These findings are in line with those of a previous study using the same stimuli and showing that musicians were more sensitive than nonmusicians to the abnormal lengthening of the penultimate syllable of trisyllabic words (Marie et al., 2011). More generally, these results support the view that deficits in children with dyslexia are linked to temporal processing of speech insofar as time-dependent variables such as VOT and duration are the most altered (Goswami et al., 2002, 2013). In this respect, the improvement found after music training in the children tested here possibly resulted from the CMT focusing on the manipulation of the temporal characteristics of sounds: rhythm and tempo for non-speech sounds and duration or voicing for speech sounds. Finally, the present results showed no deficits in pitch discrimination in dyslexics (e.g., Chobert et al., 2012), unlike previous evidence of the contrary (e.g., Baldeweg et al., 1999; Santos et al., 2007), and no improvement after CMT. However, this is to be expected since the CMT focussed on the rhythmic and temporal aspects of music training.

In sum, these results were encouraging in showing a positive effect of the CMT program on auditory-verbal variables that were not specifically trained after 3 days (18 h) of intensive intervention. While the several caveats present in this experiment (e.g., lack of an appropriate control group, multi-dimensionality of the MT program…) will be considered in the general discussion, we first present the second experiment aimed at testing the effects of this type of intervention conducted over a longer period. To this aim, the CMT program was used with a different group of children with dyslexia trained over 6 weeks. We used the same tasks as in Experiment 1 together with several standardized psychometric tests of various cognitive functions.

## Study 2: Cognitive-musical training over 6 weeks: Analysis of the effects on a battery of cognitive and speech tests

Many questions remained unanswered after Experiment 1. First, it was of importance to determine whether the effects observed after three CMT training days could be replicated in conditions more compatible with regular primary school schedule, so that it can be applied in current practice by speech therapists or other specialists. Second, it was of interest to test whether the observed effects generalized to variables directly involved in the nature of learning difficulties such as phonology, reading, or spelling. Third, one could question the sustainability of the observed effect since it would lose interest if it only proved ephemeral.

To answer these questions, we used a CMT similar in content and total duration but spread over 6 weeks and in a different context, that of a classroom of 12 dyslexic children, all of them with a main diagnosis of severe dyslexia. We took advantage of the existence, in the Marseille area, of schools providing special classrooms for dyslexic children (i.e., “CLIS-DYS”: sections for school inclusion of dyslexics). In contrast to the previous study, the experimental design involved three 6-week periods, including two untrained periods, one before (between T1 and T2) and one after (between T3 and T4) the CMT period (between T2 and T3). Measurements were taken four times, before and after each period (i.e., T1, T2, T3, and T4).

We hypothesized that the children's level of performance in auditory, phonological, and reading tasks, but not in writing and visual tasks, would specifically improve during the CMT period that is between T2 and T3. Any improvement between T1 and T2 (i.e., before the start of CMT) would suggest the influence of other, confounding factors. Moreover, the lack of significance for T4 vs. T3 comparisons would be compatible with the persistence of the beneficial effects beyond the end of CMT.

### Methods

#### Participants

A total of 12 children were grouped according to the intensity of their problems and not on age. Indeed, their age difference prompted us to work on homogeneous groups of four children based upon school criteria provided by the teaching team:

- one group that just stepped into reading: 4 boys aged 7, 9, 10, and 11 years.
- one mid-level group who did not yet reach automation in reading: two girls 9 and 10 year-old, and two 10 year-old boys.
- one group who had reached automation in reading: two girls aged 11 and two boys aged 11 and 12.

### Training protocol

All children were participating in workshops that took place during school time as detailed below, 3 h per week for 6 weeks. Within each of the 6 weeks, four interventions took place: two workshops of 1 h of CMT in full class (12 children) provided by a speech therapist and two musical workshops in smaller groups (4 children) for half an hour, including piano and percussion practice. Although they differed in mean age, the four groups basically received the same type of intervention, with similar content but with the easiest exercises for the youngest children. The content of the training was similar to Experiment 1, except for the dancing activity which was not proposed in Experiment 2.

### Assessment battery

Efficiency of the CMT method was assessed using a large battery of language and reading tests as well as other psychometric tests focusing on rhythm, auditory attention, visuo-spatial attention, sequential visual processing, phonological awareness, speed and quality of reading, audio-vocal loop in working memory. Moreover, the identification and discrimination tasks as well as the syllabic duration task used in Experiment 1 were also presented here. The tests were performed four times: at T1, 6 weeks before the start of the workshops; at T2 and T3, just before and just after the end of the workshops, respectively, and at T4, 6 weeks after the end of the workshops. Due to technical problems, the categorical perception and syllabic duration tasks were only performed twice, at T2 and T3 that is, just before and after CMT training.

### Language and cognitive tasks

Three tasks were selected from the NEPSY II Battery (“A Developmental NEuroPSYchological Assessment”; Korkman et al., 2012).

- **Auditory Attention and Response Set** Children are listening to a pre-recorded tape. Part A: when they hear the word “red” they put a red square into a box and when they hear other words, they do nothing. Part B: when they hear the word “red” they put a yellow square in the box and when they hear the word “yellow,” they put a red square in the box. When they hear the word “blue,” they put a blue square in the box. These tasks allow testing for selective and sustained attention as well as for executive function, specifically inhibition, and shifting.
- **Visuo-Spatial Attention** Children are required to cross-over as quickly as possible a specific symbol among hundreds of other symbols presented on a sheet of paper. Part B: Children are required to cross-over as quickly as possible two specific symbols among hundreds of other symbols. The level of performance is computed as the number of false alarms subtracted from the number of correct responses. Time is limited to 180 s per sheet. These tasks allow testing for perceptual attention and visuo-motor abilities.
- **Repetition of Non-sense Words** Children are asked to repeat nonsense words presented from an audiotape. These tasks allow testing for phonological encoding and decoding skills.
  Four tasks were chosen from the BALE battery (“Batterie Analytique du Langage Ecrit”; http://www.cognisciences.com) that allow testing for reading, spelling, and meta-phonological skills in French.
- **Digit Repetition Task** Children are asked to repeat sequences of digits that increase in size (forward span) or to repeat the sequences of digits in reverse order (backward span) until they make two consecutive errors. These tasks test for short-term and working memory.
- **Phonemic Fusion** Children are asked to isolate the first phonemes of two consecutively presented words and to merge them together to create a syllable [e.g., the answer for “bel animal” (“beautiful animal”) is [ba]]. Three examples are given before starting. Ten items are presented, the number of correct responses is computed and the time taken to perform the task is measured.
- **Visual Identification of Letters (Sequential Analysis)** Twenty series of 3–5 letters are presented in pairs and columns and children have to decide whether both members of the pair are similar or not.
- **Contour Discrimination** Using four different color pens, children have to highlight the contour of four intermixed stars. This task tests for perceptual discrimination.
  Finally, three standardized task were used to test for reading abilities, rhythm reproduction and writing.
- **Reading Task** (“Lecture en une Minute,” LUM; 1 min Reading Task; LMC-R Battery, Khomsi, 1999) Children are asked to read as many words as possible that are presented in a column. The number of words read in 1 min and the number of errors are recorded and the difference between the two measures gives the reading score in 1 min (LUM). This test evaluates the degree of automation in reading, a key element of reading efficiency.
- **Rhythm Reproduction Task (Stambak, 1951)** Children are asked to reproduce a set of 21 rhythmic patterns of increasing complexity that are performed by the examiner following indications on a sheet of paper. Scores are computed by counting the number of errors in the reproduction of the rhythmic patterns.
- **BHK Test (Concise Evaluation Scale for Children's Handwriting, French Version, Charles et al., 2003)** Children are asked to copy a standard text that is presented on a card for 5 min. This task tests for the quality and fluidity of handwriting. Results are scored according to 13 different criteria (such as size, regularity, variations in size or obliquity of letters, etc… ) and a separate criterion of writing speed.

### Data analysis

To allow for comparisons with Experiment 1, repeated measures ANOVAs were conducted for the categorical perception tasks that included Position or Pair and Session (T2 vs. T3) as within-subject factors. Fischer's PLSD were used for *post-hoc* comparisons. For the syllabic duration task, the ANOVA included Condition (normal vs. lengthened syllables) and Session (T2 vs. T3) as within-subject factors. Student *t*-tests were used for pre vs. post-test comparisons in the other tests. When possible, scores were transformed into standard deviation units from the norm (as provided by authors of the tests). Means, standard deviations and significance level for each individual measurement are reported in Tables 3–**5**. When relevant, effect sizes are also reported as Cohen's d (Cohen, 1988; Soper, 2015). Finally, in order to correct for multiple comparisons and considering that < 10 independent planned comparisons were computed, the significance level was set at *p* < 0.01 rather than *p* < 0.05.

**Table 3:** **Attentional processing of speech and non speech stimuli**.

**Task**	**Type of measure**	**T1 mean *(s.d.)***	**T2 mean *(s.d.)***	**T3 mean *(s.d.)***	**T4 mean *(s.d.)***	**T1/T2**		**T2/T3**			**T3/T4**		
						***T*-test**	***P*-value**	**Effect size**	***T*-test**	***P*-value**	**Effect size**	***T*-test**	***P*-value**
Auditory attention	Correct resp. (A)	32.83 (19.30)	31.33 (5.15)	45.16 (6.71)	45.25 (2.85)	0.29	0.77		**6.35**	0.001^***^	2.31	0.04	0.96
	Correct resp. (B)	29.25 (12.31)	30.16 (6.64)	38.80 (8.58)	38.50 (8.63)	0.39	0.70		**4.25**	0.01^**^	1.12	0.14	0.89
	Total (s.d. from norm)	−1.0 (0.83)	−1.00 (0.31)	−0.36 (0.54)	−0.25 (0.62)	0.01	0.99		**5.72**	0.001^***^	3.09	1.48	0.17
Visuo-spatial attention	Correct resp. (A)	19.66 (1.15)	19.91 (0.28)	2.00 (0.00)	19.91 (0.28)	0.71	0.49		1.00	0.33		1.01	0.33
	Correct resp. (B)	14.00 (3.83)	16.90 (3.20)	17.00 (2.76)	16.60 (3.11)	−**3.77**	0.003^**^	0.82	0.16	0.87		0.84	0.42
	Total (s.d. from norm)	−0.86 (1.0)	−0.50 (0.77)	−0.69 (1.12)	−0.72 (1.03)	1.77	0.10		0.50	0.62		0.11	0.91
Working memory: Digit span (s.d. from norm)	Forward (s.d. from norm)	1.51 (1.10)	1.22 (1.05)	1.13 (0.98)	0.87 (1.36)	−1.08	0.30		0.44	0.67		0.68	0.51
	Backward (s.d. from norm)	−1.07 (0.52)	−0.99 (0.47)	−0.83 (0.63)	−0.59 (0.88)	0.40	0.68		0.75	0.47		1.04	0.32
Rhythm reproduction	Errors	1.16 (3.07)	9.58 (2.81)	8.25 (2.17)	9.33 (2.08)	0.61	0.55		1.34	0.21		−1.82	0.09

Children' levels of performance were measured four times (T1, 6 weeks before CMT started; T2, just before CMT; T3, just after CMT; T4, 6 weeks after CMT ended). Mean and standard deviation or s.d. from norms (when indicated) for each task are reported. Student T-tests and p-values were computed; significant improvements are in bold (

*** p < 0.001;

** *p < 0.01). Effect sizes are also reported when relevant (Cohen's d)*.

### Results

#### A- categorical perception and syllabic duration tasks

##### Categorical perception

**Figure 3:**
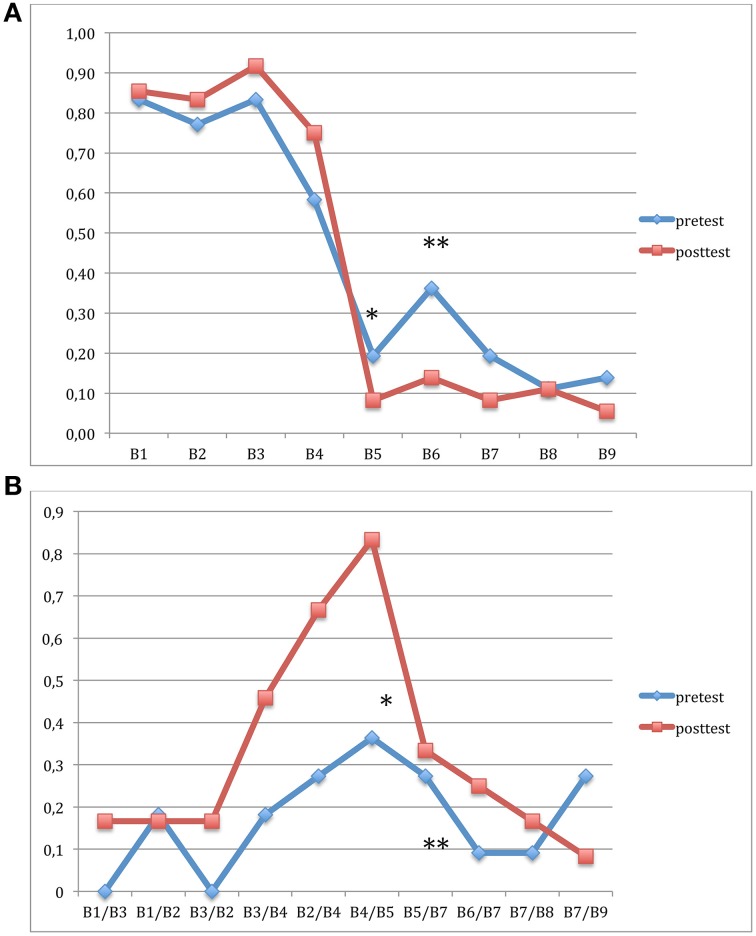
**Categorical perception in Experiment 2**. **(A)** Identification of syllables [pa] and [ba] within a 9-step acoustical continuum. The hit rate was significantly higher after than before 6-week of CMT for B5 and B6. **(B)** Discrimination: The hit rate is significantly higher for B4/B5 and B5/B7 pairs (i.e., around the categorical boundary) after than before CMT. ^*^p < 0.05; ^**^p < 0.01.

For the identification task (Figure 3A), results revealed that both the main effect of Position and the Session × Position interaction were significant [*F*_(1, 11)_ = 35.58, *p* < 0.001 and *F*_(8, 88)_ = 2.50, *p* < 0.01, respectively] but the main effect of Session was not significant (*F* < 1). *Post-hoc* comparisons showed that the improvement from T2 to T3 was significant for positions B5 and B6 (i.e., around the phonemic [ba]-[pa] boundary with *p* < 0.03 and *p* < 0.004, respectively).

For the discrimination task (Figure 3B), the main effect of Pair was significant [*F*_(1, 11)_ = 4.46, *p* < 0.001] and the main effect of Session was marginally significant [*F*_(1, 11)_ = 2.88, *p* < 0.11]. The Session × Position interaction was not significant [*F*_(7, 77)_ = 1.39, *p* = 0.21]. However, *post-hoc* analyses revealed that the improvement from T2 to T3 was significant for the B4/B5 pair (*p* < 0.02) and for the B5/B7 pair (*p* < 0.004).

##### Syllabic duration task

The main effect of Condition was not significant [*F*_(1, 11)_ = 1.50, *p* = 0.24] so that lengthened words were not processed differently than normally spoken words. The main effect of Session was only marginally significant [*F*_(1, 11)_ = 2.42; *p* < 0.14]. The Session × Condition interaction was not significant (*F* < 1) and *post-hoc* analyses also revealed marginal improvements between T2 and T3 for both normally spoken words (*p* = 0.10) and words with syllabic lengthening (*p* = 0.08).

#### B- attentional processing of speech and non speech stimuli

##### Auditory attention

Results for the two auditory attention subtests from the NEPSY battery showed that total performance (*t* = −5.72, *p* < 0.001) and performance in both subtests (A: *t* = −6.35, *p* < 0.001) and (B: *t* = 4.25, *p* < 0.01) were improved from T2 to T3 with no decrease from T3 to T4 (all *p* > 0.15) and with no difference between T1 and T2 (all *p* > 0.70; see Table 3).

##### Visuo-spatial attention

No significant improvement was found from T2 to T3, but performance improved from T1 to T2 when children received no treatment (T1-T2: *t* = 3.77, *p* < 0.003; see Table 3).

##### Digit repetition task from the battery BALE

No significant improvement was found whatever the period considered (all *p* > 0.30, see Table 3).

##### Reproduction of motor rhythmic *sequences*

No significant differences were found whatever the period considered (all *p* > 0.10, see Table 3).

#### C- phonological and reading tasks

##### Pseudo-word repetition

**Figure 4:**
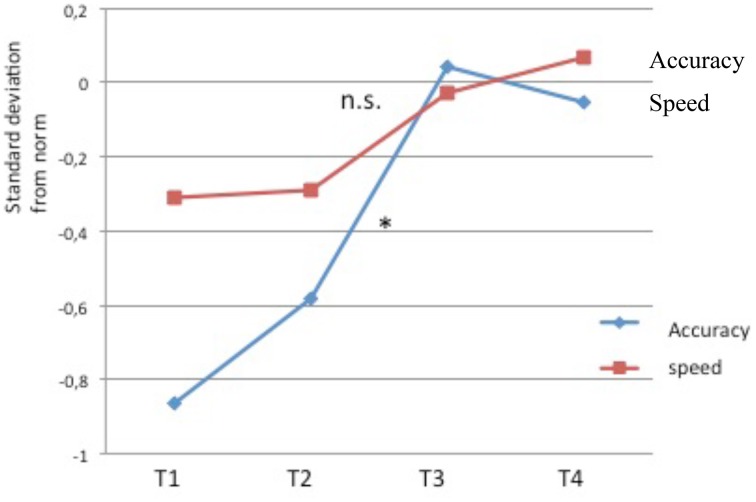
**Phonemic fusion task (in standard-deviations from age norm)**. Evolution of the level of performance across time. Significant improvements are found for accuracy (^*^p < 0.05) but not for speed (ns: non significant).

Results showed improvements from T2 to T3 (*t* = −2.56, *p* < 0.026), no decrease from T3 to T4 (*p* > 0.15) and no difference between T1 and T2 (*p* > 0.80, see Table 4 and Figure 4).

**Table 4:** **Phonological and reading tasks**.

**Task**	**Type of measure**	**T1 mean *(s.d.)***	**T2 mean *(s.d.)***	**T3 mean *(s.d.)***	**T4 mean *(s.d.)***	**T1/T2**		**T2/T3**			**T3/T4**	
						***T*-test**	***P*-value**	***T*-test**	***P*-value**	**Effect size**	***T*-test**	***P*-value**
Pseudo-word repetition	Pseudo-word span	21.08 (8.83)	21.5 (6.20)	24.75 (6.16)	26.50 (6.15)	0.21	0.84	**2.57**	0.03^*^	0.52	1.48	0.17
Reading in 1 min (LUM)	Nb items read (*s.d.* from norm)	−2.24 (1.32)	−2.12 (1.49)	−1.66 (1.59)	−1.64 (1.64)	1.34	0.20	**5.59**	0.001^***^	0.29	0.20	0.85
Phoneme fusion (s.d. from norm)	Phonemic fusion score	−0.86 (0.96)	−0.58 (0.86)	0.04 (0.73)	−0.05 (0.80)	1.04	0.32	**2.90**	0.01^**^	0.78	0.70	0.50
	Time phoneme fusion	−0.31 (1.19)	−0.29 (1.15)	−0.02 (1.13)	0.06 (0.86)	0.32	0.75	1.94	0.07		0.46	0.65

Children' levels of performance were measured at T1 (6 weeks before CMT started) at T2 (just before CMT), at T3 (just after CMT), and at T4 (6 weeks after CMT ended). Mean and standard deviation or s.d. from norm (whenever indicated) are reported for each task. Student T-tests and p-values were computed and significant improvements are in bold (

*** p < 0.001;

** p < 0.01;

* *p < 0.05). Effect sizes are also reported when relevant (Cohen's d)*.

##### Reading in 1 min (Khomsi test)

Results showed improvements from T2 to T3 (*t* = −5.59, *p* < 0.001) with no significant decrease from T3 to T4 (*p* > 0.80) and no significant difference between T1 and T2 (*p* > 0.20, see Table 4).

##### Phoneme fusion (BALE)

Results showed an improvement from T2 to T3 for accuracy (*t* = −2.90, *p* < 0.01), with only a tendency for speed (*t* = 1.94, *p* = 0.07). No decrease was found from T3 to T4 whether for accuracy or for speed (all *p* > 0.40) and no difference was found between T1 and T2 (all *p* > 0.30; see Table 4 and Figure 4).

#### D- visual and writing tasks

##### Comparison of letter strings (BALE)

The improvement from T2 to T3 was significant for speed (*t* = 3.41, *p* < 0.006) but only marginally significant for accuracy (*t* = −1.78; *p* = 0.10). No significant decrease was found from T3-T4 (*p* > 0.40 in both cases) and no significant difference between T1 and T2 (*p* > 0.20 in both cases, see Table 5).

**Table 5:** **Visual and writing abilities**.

**Task**	**Type of measure (*(s.d.)* from norm)**	**T1 mean *(s.d.)***	**T2 mean *(s.d.)***	**T3 mean *(s.d.)***	**T4 mean *(s.d.)***	**T1/T2**		**T2/T3**			**T3/T4**			
						***T*-test**	***P*-value**	**Effect size**	***T*-test**	***P*-value**	**Effect size**	***T*-test**	***P*-value**	**Effect size**
Letter-sequence comparison	Score	−1.27 (1.74)	−2.21 (2.98)	−0.55 (0.90)	−0.46 (1.07)	1.01	0.33		1.78	0.10		0.56	0.59	
	Time	−1.54 (1.24)	−1.36 (1.22)	−0.40 (0.50)	−0.28 (0.72)	1.31	0.21		**3.41**	0.006^**^	1.02	0.84	0.42	
Contour discrimination	Nb correct contours	−4.97 (6.25)	−1.44 (3.31)	−1.44 (3.84)	−1.44 (3.84)	**2.55**	0.03^*^	0.70	n.a.			n.a.		
Writing test BHK	Score quality	−1.84 (2.37)	−1.70 (2.36)	−1.19 (2.15)	−1.36 (2.51)	0.43	0.67		1.08	0.30		0.35	0.74	
	Speed	−1.65 (0.61)	−1.55 (0.60)	−1.67 (0.67)	−1.28 (0.80)	0.98	0.18		1.46	0.17		**2.17**	**.05**	**0.52**

Children' levels of performance were measured at T1 (6 weeks before CMT started) at T2 (just before CMT), at T3 (just after CMT), and at T4 (6 weeks after CMT ended). Standard deviation from norm for each task are reported. Student T-tests and p-values were computed and significant improvements are in bold (

** p < 0.01;

* *p < 0.05). Effect sizes are also reported when relevant (Cohen's d). n.a.: not available*.

##### Contour discrimination (BALE)

**Figure 5:**
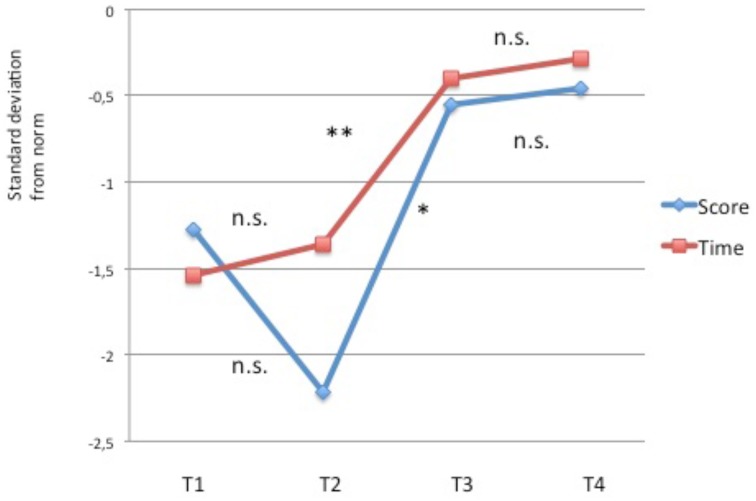
**Comparison of letter strings (in standard deviations from age-norm)**. Evolution of the level of performance across time. Significant improvements from T2 to T3 for speed (Time: ^**^p < 0.01; ^*^p < 0.05) but marginally significant for accuracy (score: ^*^p < 0.05).

The improvement from T1 to T2 was significant (*t* = 2.55, *p* < 0.03) with no change from T2 to T3 or from T3 to T4 (see Table 5 and Figure 5).

##### BHK (writing)

The improvement from T3 to T4 was marginally significant (*t* = −2.17, *p* < 0.05) with no change from T2 to T3 or from T1 to T2 (*p* > 0.15, see Table 5).

Finally, correlational analyses between the different tasks did not reveal any significant results when the significance level is set at *p* < 0.01.

### Discussion

The first aim of Experiment 2 was to determine whether results similar to Experiment 1 would be found when CMT was spread over time. The second aim was to assess whether or not this effect also extended to standardized psychometric tests known to be sensitive to learning difficulties encountered by children with dyslexia. Overall, these two objectives were reached. As in Experiment 1, results revealed improved categorical perception of syllables after the period of CMT (significant Session × Condition interaction in both experiments). While improvements were found both for the quality of intra-categorical perception and for inter-categorical boundary in Experiment 1 (i.e., at B2, B6 and B9), the improvement was mainly found for inter-categorical boundary in Experiment 2 (i.e., at B5 and B6). Nevertheless, in both experiments and in line with previous results in younger and older adults (Bidelman et al., 2014), music training seemed to positively influence categorical perception in children with dyslexia. Turning to the perception of syllabic duration, the Session by Condition was marginally significant in both experiments. While the effect of CMT was larger for lengthened than normally spoken words in Experiment 1, the perception of both types of words was marginally improved after the CMT in Experiment 2. Thus, overall results were similar in both Experiments, showing that the CMT program positively influenced categorical perception and the temporal aspects of speech processing. Importantly, these effects were found in Experiment 2 when the CMT program was spread over time and, consequently, more compatible with standard speech therapy practice.

Results of standardized psychometric tests showed that several aspects of the children's behavior that were directly targeted by the CMT program specifically improved during the period of music training. This was clearly the case for auditory attention, pseudo-word repetition, reading words in 1 min, phonological awareness (phonemes fusion), and comparison of letter strings. Specifically, for auditory attention, results showed almost 15% gain in selective attention, 10% in divided attention, and 20% in total score from T2 to T3. Importantly, these improvements persisted in the following period without further training (from T3 to T4: no significant decrease in level of performance). Turning to the reading tests that were probably the most interesting due to their strong relationship to academic performance, the improvement after CMT was almost one standard deviation from the norm, moving from a score lower to -2 s.d. to nearly -1 s.d. from controls scores. Similar to results for auditory attention, pseudo-word repetition and phoneme fusion (scores), these reading improvements persisted unchanged for 6 weeks after the end of the CMT period, thereby pointing to the durability of the CMT program.

Equally important, the effect of the CMT program was not significant between T2 and T3 on the control variables for which we did not *a priori* predict an effect of this type of treatment, such as visuo-spatial attention, contour discrimination, and writing efficiency. However, some effects, such as the significant improvement between the untrained period from T1 to T2 for visual attention and contour discrimination and from T3 to T4 for writing efficiency, were unexpected and may result from mere repetition effects. They need to be replicated and examined in further experiments. More surprisingly in view of several results in the literature showing strong links between rhythmic and linguistic abilities (Overy, 2000, 2003; Przybylski et al., 2013; Slater et al., 2013; Bishop-Liebler et al., 2014; Flaugnacco et al., 2014; Weiss et al., 2014) and in view of the strong focus of the CMT program on the temporal association between sensory input and motor activities, we found no significant improvements in memory span and in the rhythm reproduction tasks. While these null findings are difficult to interpret, it may be that the specific rhythmic test used here was not best adapted to capture potential improvements or that training temporal processing may generalize to other cognitive functions without perceptible improvement on the trained function itself. Finally, correlational analyses between the different tasks did not reveal any significant results.

## General discussion

Overall, our results provide convincing arguments in favor of using musical rehabilitative materials with children with dyslexia. The different aspects of the CMT program were specifically designed to improve sound perception, multiple aspects of temporal processing, and the integration of information from different sensory and motor modalities. In this respect, further experiments are needed to try disentangling the effects of these different components or, at least, to specify the weight of their respective contribution. Moreover, other characteristics of the training were: progressive learning, repetition of exercises, multiple modalities, and small-group workshops. Altogether, our results are in line with the repeated and longstanding observation from teachers, clinicians and scientists, that music in general, and perhaps more specifically learning an instrument, interfere positively with basic scholastic skills. Reading is the area that has been most directly tested probably as the most likely to have a direct impact on academic success. It was thus encouraging to find a significant impact of the CMT on reading ability.

The model most often put forward to account for a possible effect of music on cognitive development, specifically the acquisition of reading, calls upon a possible analogy between music and language. Many authors, most notably Patel (2003, 2011), have discussed this point, noting, in particular, that music and speech share many features such as the sequence of sounds, an alphabet that represents them, and a specific syntax. Patel (2011) hypothesized that music leads to adaptive brain plasticity of the same neural networks which are otherwise involved in language processing. More recently, it has been proposed to rather conceive music and language as sharing common cognitive ressources, especially attentional and memory ressources which could be equally recruited by music and language (Rogalsky et al., 2011; Perruchet and Poulin-Charronnat, 2013).

Concerning more specifically the topic of dyslexia, our results stand in favor of a role of music training in improving the phonological deficit widely recognized as causal to the reading problems (Ramus, 2004), although others have questioned such an interpretation (Morais et al., 2010). In an influential theory of the mechanism underlying dyslexia, Goswami (Goswami et al., 2002, 2013) proposed that the dyslexics' cognitive system is specifically unable to process stimuli occurring at a frequency (frequency modulation) of the order of 2–10 cycles per second, which is the approximate frequency of syllables. As a consequence, dyslexics encounter difficulties capturing the segmental features of words and phrases. Thus, a developmental defect in processing the amplitude envelope of speech could lead to defective development of the phonological system in aspects related to the pace and patterns of intonation (prosody). In fact, children with dyslexia have been found to perform poorly on tasks of rhythmic perception and perception of musical meter (Huss et al., 2011). These observations have led researchers to propose rhythmic stimulation as a treatment for these aspects of the dyslexic deficit, and by extension, for the dyslexic disorder itself (Flaugnacco et al., 2015).

One of the strongest effect of the CMT program was on the attentional tests, in particular, the two subtests of the NEPSY auditory attention battery assessing selective and divided attention. An overall improvement of 20% over the 6 weeks of training is a successful outcome, in particular since this effect persisted after a 6 weeks untrained period. A recent brain imaging study (Heim et al., 2015) showed that dyslexics who received three different types of remediation (based on phonology, on attention, or on reading training) ultimately had similar pattern of improvement in terms of brain activation (specifically, an increase in activity in the lower left temporal region). It is thus conceivable that the improvements we have seen in our study on reading and phonology tasks are epiphenomenons, reflecting an impact on the attention system. We could not, however, find evidence of any correlations between the degree of improvement in phono-lexical tasks and attentional tasks. While the improvement was significant on tests of auditory attention, no significant changes were found on tests of visual attention, contrary to what one might expect if the CMT effect was operating through general attentional mechanisms. The fact remains that a positive effect on attention certainly contributed to the overall improvement even if the multi-faceted aspects of the CMT program preclude from concluding that this was the sole factor responsible for the improvements.

The cross-modal aspects of the CMT program may account for the effects found for tasks requiring sharing information between different modalities, such as reading and sequence comparison of letters as well as the phonological task, if one considers that such tasks require mandatory exchanges of information between the acoustic representation of phonemes that can be stored in auditory regions of the left temporal lobe, and the lower frontal areas, involved in phonological processing (Boets et al., 2013). Similarly, categorical perception also probably requires the involvement of structures such as the left frontal premotor areas that was found to be activated in dyslexics during a categorical perception task in a functional MRI experiment (Dufor et al., 2009). Finally, studies of brain plasticity in non musicians have shown that music training may have the largest effects on brain anatomy and function when it combines sensory and motor training (Lappe et al., 2008, 2011). The recent literature on dyslexia and related learning deficits converge to show that the main structural differences in the brains of dyslexics compared to standard brains lie in the nature, integrity and directionality of certain hemispheric white matter bundles. These differences are present before learning to read (Saygin et al., 2013) and therefore can not be the result of a lack of experience with written language (although this may contribute as shown by studies of illiterates: Thiebaut de Schotten et al., 2014). These association bundles are also altered in musicians, both instrumentalists and singers (Halwani et al., 2011) and change in children after only a few months of musical training (Hyde et al., 2009). Although DTI is often regarded as a very indirect measure of white matter integrity (Jones et al., 2013), it remains that the same white matter bundles considered as the hallmark of the dyslexic brain are modified by musical training. Although strictly speculative, this observation deserve future investigation.

Using fMRI, Blau et al. (2009) have shown that dyslexics are characterized by poor integration of oral and written codes of the same phoneme. When these codes are congruent, temporal areas are less activated by combined visual-auditory processing than in controls; when the oral and written codes are incongruent, temporal areas are more strongly activated than in controls. An interpretation in terms of aberrant crossmodal integration can also potentially explain other clinical conditions such as dysgraphia, in which children hardly associate phonemes with their written form, or dyscalculia, in which they hardly relate numerical terms with the mental representation of the corresponding quantity (Noël et al., 2013).

Finally, we shall consider some of the potential caveats of the present study, specifically the absence of a control group, trained for comparison with another, already proven training method comparable in duration and cognitive load. Besides the obvious difficulty of finding such an ideal comparison group, our training protocol in Experiment 2 partly fulfills this objective, providing evidence for the selectivity of improvements, at least for some tasks, during the training period (T3 vs. T2) compared to the non-trained period (T2 vs. T1). Thus, potential motivational bias (so-called Hawthorne effect) can reasonably be ruled out by this design. Overall, we remain convinced that intra-subject approaches and disease progression models could be more convenient than larger samples for building comparison groups (since it is often difficult to recruit large samples in clinical settings). By having a pre-test/post-test design, we examined group improvement within subjects rather than between-subjects. With a lower number of individuals, this may have resulted in more extraneous effects due to individual differences. A second weakness is that improvement of specific cognitive mechanisms can not be distinguished from a purely attentional effect. In face of the consistency of improvement on attentional variables, the possibility of an exclusive or largely predominant impact of attention on executive processes can not be ruled out. Further experiments are needed to compare our results to the effect of a strictly attentional training protocol.

## Conclusion

In a recent yet already acclaimed book, suggestively entitled “The Dyslexia Debate,” Elliott and Grigorenko (2014), two eminent specialists of the topic, provided arguments questionning the usefulness, or even reality, of the concept of dyslexia. This is based on the observation that various theories are proposed in the literature, none of them being entirely satisfactory and that most of the existing remediation methods are pedagogic rather than properly therapeutical. Accordingly, our conviction is that although the reality of a biological entity is indeed unquestionable, the multifaceted and kaleidoscopic clinical appearance of dyslexia, as suggested by the recent changes made to the DSM classification (APA, 2013), may lead to consider using multiple-component treatments, such as the ones offered by music training, rather than focusing on one single cognitive mechanism as in classical phonological training methods.

Music training may provide an ideal tool for such a new perspective: it allows considering each one of the multiple facets of dyslexia as a potential target to be improved. In this respect, music training may be one of the most complete and rational ways of treating dyslexia. Whatever the exact mechanism(s) subserving the observed improvements, their occurrence after relatively short sessions of musical training opens interesting avenues for future research as well as practical applications. First, our results suggest that several cognitive functions, including reading but not only, may be improved by adding a musical content to classical speech therapy and remediation of dyslexia. Our view is that such training could usefully complement more classical methods, in particular when they have been used extensively but children still need reeducation. Second, as others have also noted (Heim et al., 2015), the improvement may depend upon two main features of the CMT method; an intensive training and that this training is given collectively to small groups of children. Finally, our results open new avenues for future research. For instance, it would be of interest to include recording of electrophysiological or neuroimaging data, to assess the brain changes underlying the observed improvements. Also, direct comparisons with other remediation methods could provide important additional understanding of the exact nature of the improved processes, for example by comparing musical training to more specific attentional or phonological training. Finally, testing the hypothesis of impaired connectivity in other neurodevelopmental disorders such as dyscalculia (Srinivasan and Bhat, 2013) would certainly contribute to enrich the “The Dyslexia Debate” (Elliott and Grigorenko, 2014).

### Conflict of interest statement

The authors declare that the research was conducted in the absence of any commercial or financial relationships that could be construed as a potential conflict of interest.
